# Prognosis Prediction of Cardiovascular Event With Glucose‐Albumin Ratio on Patients With Cancer and Prescribed With Anthracycline

**DOI:** 10.1002/cam4.70471

**Published:** 2024-12-11

**Authors:** Iokfai Cheang, Ying Li, Xu Zhu, Ziqi Chen, Qing‐Wen Ren, Mei‐Zhen Wu, Xin Xu, Hung‐Fat Tse, Kai‐Hang Yiu, Xinli Li

**Affiliations:** ^1^ State Key Laboratory for Innovation and Transformation of Luobing Theory, Department of Cardiology The First Affiliated Hospital of Nanjing Medical University, Jiangsu Province Hospital Nanjing China; ^2^ Division of Cardiology, Department of Medicine The University of Hong Kong ‐ Shenzhen Hospital Shenzhen China; ^3^ Cardiology Division, Department of Medicine The University of Hong Kong, Queen Mary Hospital Hong Kong China

**Keywords:** anthracycline, biomarker, cancer, cardiotoxicity, chemotherapy, GAR, glucose‐albumin ratio, prognosis

## Abstract

**Aims:**

This study aimed to investigate the clinical value of the glucose‐albumin ratio (GAR) in predicting the prognosis of cancer patients prescribed anthracycline‐based chemotherapy.

**Methods:**

We included cancer patients who underwent anthracycline‐based chemotherapy, drawn from the population‐based cohort Clinical Data Analysis Reporting System of Hong Kong between January 2000 and December 2019. Demographics, medical history, baseline laboratory, and metabolic indicators, including GAR, were collected. We employed random survival forests (RSF) with the variable importance (VIMP) method to rank the importance of these variables. Cox proportional hazards regression was used to assess the association between GAR levels and event risks.

**Results:**

A total of 18,700 patients were included in the analysis. The top 2 factors for predicting overall cardiovascular event risk were GAR and fasting blood glucose (FBG). Our results revealed that a higher GAR was significantly associated with poorer cardiovascular prognosis in patients with cancer prescribed with anthracycline‐based chemotherapy. Compared to the lowest quartile, higher GAR levels were significantly associated with increased risk of all‐cause mortality, major adverse cardiovascular events, hospitalization of heart failure, and cardiovascular mortality regardless of the adjustments (all *p* trend < 0.001).

**Conclusion:**

GAR is a potential biomarker for predicting the prognosis of cancer patients undergoing anthracycline‐based chemotherapy. Monitoring GAR levels before and during treatment may help identify patients at higher risk of adverse outcomes, facilitating personalized treatment strategies and improving clinical management.

## 1 Introduction

Cancer remains one of the leading causes of mortality worldwide, necessitating continuous efforts to identify reliable prognostic markers that can aid in patient management and treatment planning [1, 2]. Anthracyclines, a class of chemotherapy agents, have been widely used in the treatment of various cancers due to their potent antitumor activity [3]. However, anthracycline‐based chemotherapy is associated with a range of side effects and variable treatment outcomes, highlighting the importance of personalized medicine approaches to optimize patient care [4].

Cardiovascular disease (CVD) represents a significant concern in the management of cancer patients, particularly those undergoing anthracycline‐based chemotherapy, due to the potential cardiotoxic effects of these agents. While anthracyclines possess potent antitumor properties, their use is often limited by adverse cardiovascular events, including cardiomyopathy, heart failure (HF), and arrhythmias [5]. While the presence of metabolic comorbidities, such as diabetes and pre‐diabetes, could further elevate the risk of HF and adverse outcomes [6, 7], comprehensive cardiovascular monitoring, aggressive management, and a multidisciplinary treatment approach are crucial in mitigating these risks and improving patient outcomes [8]. Thus, there is a critical need to identify reliable prognostic markers that can aid in predicting cardiovascular risk and optimizing patient care in this vulnerable population.

Recent studies have suggested that metabolic and nutritional status indicators can provide valuable insights into cancer patient prognosis and treatment response [9, 10, 11]. The glucose‐albumin ratio (GAR), calculated by dividing fasting blood glucose levels by serum albumin levels, has emerged as a potential biomarker reflecting both metabolic and nutritional states. Elevated GAR has been linked to adverse outcomes in various clinical settings by reflecting the nutrition status [12, 13].

Given the dual impact of cancer and anthracycline therapy on cardiovascular health, understanding the prognostic significance of GAR in this context is of paramount importance. This study aims to evaluate the prognostic value of GAR in predicting cardiovascular events in patients with cancer who are prescribed anthracycline‐based chemotherapy. By elucidating the association between GAR and cardiovascular outcomes, we intend to offer clinicians a novel and practical tool for risk stratification and personalized management of patients at risk of cardiotoxicity from anthracycline treatment.

## 2 Method

### 2.1 Study Population

The Clinical Data Analysis Reporting System (CDARS) is a comprehensive database managed by the Hong Kong Hospital Authority (HA), capturing clinical data from both inpatient and outpatient settings since January 01, 1993. With its extensive coverage, the HA serves over 80% of the population of approximately 7.5 million Hong Kong citizens (source: HA Statistics, https://www3.ha.org.hk/data/HAStatistics, Accessed June 2, 2024). Patient data in CDARS are de‐identified, ensuring anonymity, and are approved for research use by the University of Hong Kong institutional review board and the West Cluster of HA (UW 21–270), with waived informed consent due to the retrospective and anonymous nature of the data.

Our study included chemotherapy‐naive adult patients diagnosed with solid or hematological malignancies who received anthracycline‐based chemotherapy (ACT) between January 1, 2000, and December 31, 2019. The index date was defined as the initiation of anthracycline treatment. Patients with a life expectancy of fewer than 14 days were excluded. Cumulative anthracycline doses were calculated based on doxorubicin equivalents, adjusted for body surface area using established conversion factors for different anthracyclines [4, 7].

### 2.2 Data Collection

Baseline characteristics of the study population were comprehensively collected from the CDARS, encompassing demographic information such as age and sex, cancer diagnoses including etiology and metastasis status, and pertinent comorbidities such as coronary artery disease (CAD), heart failure (HF), atrial fibrillation (AF), hypertension (HTN), and type 2 diabetes mellitus (DM). Laboratory test results, including creatinine, red cell distribution width (RDW), neutrophil count (NEU), fasting blood glucose, HbA1c, albumin levels, etc., were obtained. For each participant, the ratio of red cell distribution width to albumin (RAR), neutrophil count to albumin (NAR), fasting glucose to albumin (GAR), and HbA1c to albumin (HAR) were calculated to evaluate the metabolic and nutritional status.

Additionally, records of drug prescriptions, including angiotensin‐converting enzyme inhibitors/angiotensin receptor blockers (ACEI/ARB), statins, anthracyclines, and other chemo‐regimen, were captured. Laboratory investigations, hospitalization details, and outpatient visits were also prospectively documented within CDARS. Diagnoses were coded using the International Classification of Diseases, Ninth (ICD‐9), and Tenth Revision (ICD‐10), ensuring uniformity and accuracy in data representation (summarized in Table S1). Previous studies have reported a high level of coding accuracy within CDARS [7, 14].

### 2.3 Outcomes Ascertainment

The primary endpoint of the study was defined as major adverse cardiovascular events (MACE), comprising CVD mortality and hospitalization due to heart failure (HHF), whichever occurred first. Secondary outcomes included cardiovascular mortality, HHF, and all‐cause mortality.

To ascertain outcomes, linked mortality records were retrieved and identified using the ICD‐10 code for cardiovascular causes (I00‐I99). Instances of HHF were identified through relevant hospital discharge codes. Patients were followed up from the day after the index date until the occurrence of outcomes, death, or the last date of data collection (August 01, 2022), whichever came first. This comprehensive approach ensured accurate and complete capture of relevant endpoints, facilitating robust analysis and interpretation of study findings.

### 2.4 Statistical Analyses

Descriptive statistics were utilized to present continuous variables, with means and standard deviations (SDs) for normally distributed data and medians with interquartile ranges [IQRs] for non‐normally distributed data. Categorical variables were expressed as numbers and percentages.

Variable importance (VIMP) analysis, derived from random survival forest analysis, elucidated the relative significance of various clinical factors in predicting outcomes. Cox proportional hazards regression was performed for estimation of hazard ratios (HR) with 95% confidence interval (CI) to assess the association between GAR (as quartiles, with the lowest quartile as reference) and clinical outcomes, adjusting for key covariates including age, sex, body mass index, estimated glomerular filtration rate, aspartate aminotransferase, HTN, DM, AF, CAD, tumor type, ACEI/ARB, statin use, and cumulative dosing of anthracyclines, among others. Additionally, restricted cubic spline (RCS) regression models, with knots strategically placed at the 10th, 50th, and 90th percentiles of GAR, elucidated the potential non‐linear relationship between GAR levels and hazard ratios for adverse events. Subgroup analyses were performed to explore potential effect modifiers such as age, sex, baseline comorbidities, cumulative dosing of anthracyclines, other chemotherapy regimens, and overweight/obesity status. Furthermore, receiver operating characteristic (ROC) analysis was conducted at various time points (3‐, 5‐, 10‐, and 15‐) to evaluate the predictive performance of GAR in discerning cardiovascular risk in this patient population.

A sensitivity analysis was conducted to evaluate the improvement in risk prediction by adding the GAR to fully adjusted models. Furthermore, analysis was conducted by excluding participants who died within 1 year of follow‐up to assess the robustness of the glucose‐to‐albumin ratio (GAR) as a predictor for various outcomes in cancer patients treated with anthracyclines.

R software (version 4.1.0; The R Foundation for Statistical Computing) was used for data analysis. A two‐sided P‐value of less than 0.05 was considered statistically significant.

## 3 Results

### 3.1 Baseline Characteristics

**FIGURE 1:**
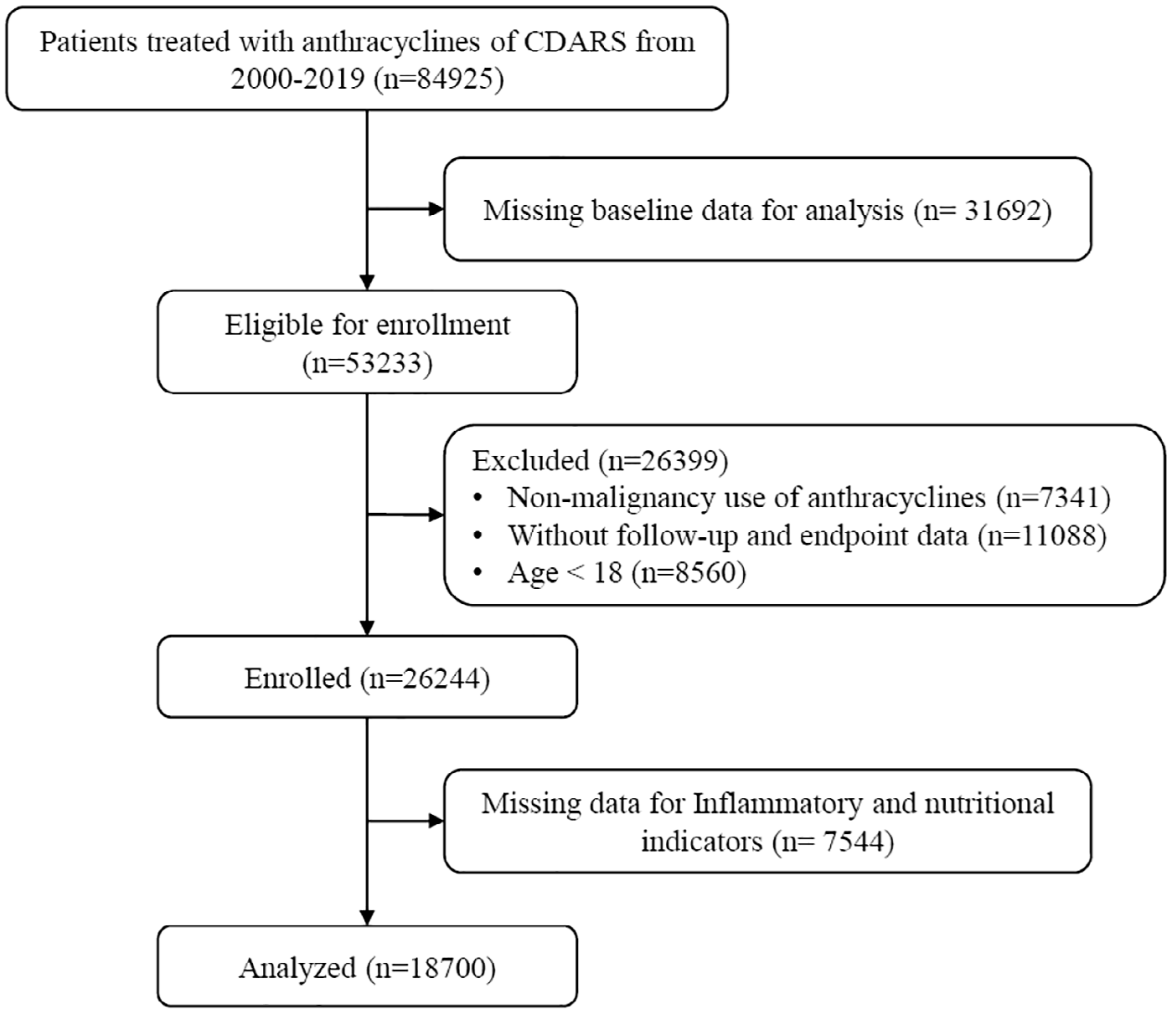
Study Flowchart.

Patient demographics and characteristics for the 18,700 patients with cancer who were prescribed anthracycline included in this study (Figure 1). The median age of the study population was 62 years old (IQR: 50–73), with 53.6% of patients being male. The median GAR was 0.17 (IQR: 0.13–0.24). During a median follow‐up of 8.7 years, 10,359 (55.4%) patients died, and 1,631 (8.7%) cases of MACE occurred. Table 1 presents the baseline characteristics of patients stratified by the occurrence of MACE.

**TABLE 1:** Baseline Characteristics.

	All Patients	MACE	
		No	Yes
Participants, No.	18,700	17,068	1632
Male (%)	10,014 (53.6)	9059 (53.1)	955 (58.5)
Age, years old	62.00 [50.00, 73.00]	61.00 [49.00, 72.00]	74.00 [64.00, 81.00]
Body Mass Index, kg/m^2^	24.39 (4.37)	24.36 (4.36)	24.71 (4.46)
Baseline Comorbidity (%)			
Atrial Fibrillation	440 (2.4)	268 (1.6)	172 (10.5)
Hypertension	2085 (11.1)	1648 (9.7)	437 (26.8)
Diabetes mellitus	1415 (7.6)	1096 (6.4)	319 (19.5)
Coronary Artery Disease	310 (1.7)	209 (1.2)	101 (6.2)
ACEI/ARB (%)	2234 (11.9)	1738 (10.2)	496 (30.4)
Statin (%)	2666 (14.3)	2248 (13.2)	418 (25.6)
Etiology (%)			
Hematologic diseases	5664 (30.3)	5316 (31.1)	348 (21.3)
Malignant neoplasm of bladder	4737 (25.3)	4055 (23.8)	682 (41.8)
Malignant neoplasm of breast	2121 (11.3)	2043 (12.0)	78 (4.8)
Nonspecific neoplasm	6178 (33.0)	5654 (33.1)	524 (32.1)
Anthracycline Type (%)			
Daunorubicin	5157 (27.6)	4940 (28.9)	217 (13.3)
Doxorubicin	1216 (6.5)	1119 (6.6)	97 (5.9)
Epirubicin	3389 (18.1)	3205 (18.8)	184 (11.3)
Mitoxantrone	1427 (7.6)	1367 (8.0)	60 (3.7)
Idarubicin	7511 (40.2)	6437 (37.7)	1074 (65.8)
Cumulative dosing, mg/m2	162.88 [92.00, 332.34]	162.28 [89.30, 319.27]	164.70 [94.33, 344.67]
Other chemo‐regimen (%)	8333 (44.6)	7950 (46.6)	383 (23.5)
Laboratory parameters			
AST, U/L	24.00 [18.00, 34.00]	24.00 [18.00, 33.40]	25.00 [19.00, 37.00]
eGFR, ml/(min × 1.73m^2^)	82.66 [66.98, 99.35]	84.03 [68.49, 100.38]	68.54 [51.35, 84.42]
NEU, 10^9^/L	4.80 [3.40, 6.85]	4.77 [3.40, 6.80]	5.10 [3.70, 7.40]
RDW, %	13.90 [13.00, 15.50]	13.80 [13.00, 15.50]	14.00 [13.10, 15.40]
FBG, mg/dL	6.36 [5.20, 8.70]	6.25 [5.20, 8.50]	7.90 [5.90, 11.40]
HbAlc, %	5.70 [5.40, 6.52]	5.70 [5.40, 6.50]	6.10 [5.50, 7.30]
ALB, g/L	39.00 [36.00, 42.00]	39.00 [36.00, 42.00]	38.01 [35.00, 41.00]
NAR	0.12 [0.09, 0.18]	0.12 [0.09, 0.18]	0.14 [0.10, 0.20]
RAR	0.36 [0.32, 0.43]	0.36 [0.32, 0.43]	0.37 [0.33, 0.43]
GAR	0.17 [0.13, 0.24]	0.16 [0.13, 0.23]	0.21 [0.15, 0.31]
HAR	0.15 [0.13, 0.18]	0.15 [0.13, 0.18]	0.16 [0.14, 0.20]

*Note:* Values are median (interquartile range) or *n* (%).

Abbreviations: ACEI/ARB, angiotensin converting enzyme inhibitors/angiotensin‐receptor blockers; ALB, albumin; AST, Aspartate aminotransferase; eGFR, estimated glomerular filtration rate; FBG, fasting blood glucose; GAR, glucose‐to‐albumin ratio; HAR, Hemoglobin A1c to albumin ratio; HbAlc, hemoglobin A1c; NAR, neutrophil‐to‐albumin ratio; NEU, neutrophil; RAR, red blood cell distribution width‐to‐albumin ratio; RDW, red blood cell distribution width.

### 3.2 Ranked Variable Importance (VIMP) of the Metabolic and Nutritional Parameters

**FIGURE 2:**
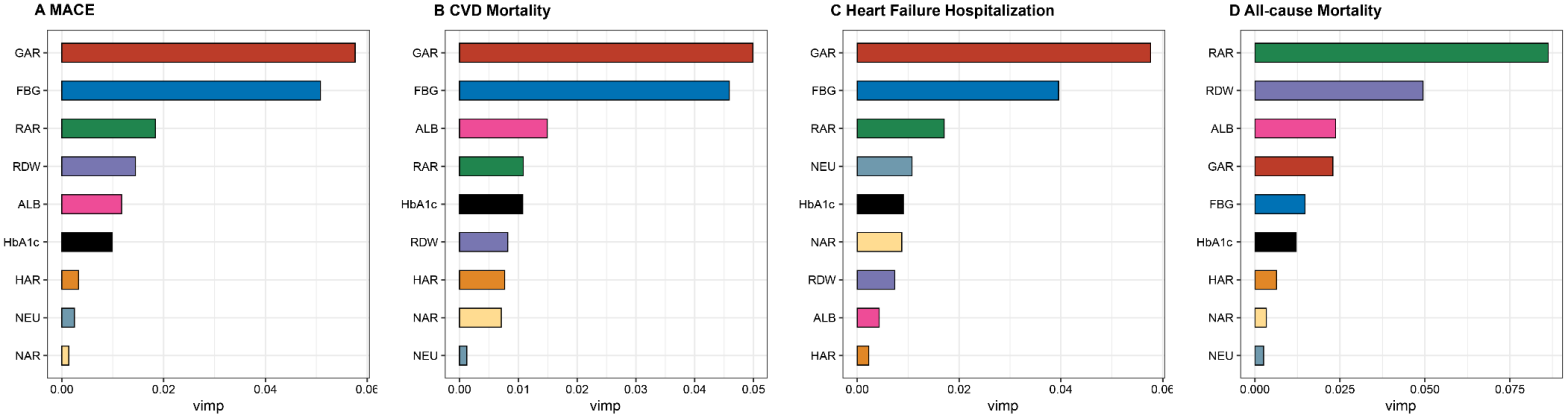
The variable importance of (A) major adverse cardiovascular events (MACE), (B) cardiovascular disease (CVD) mortality, (C) hospitalization due to heart failure (HHF), and (D) all‐cause mortality.

To evaluate the individual prognostic value of each candidate parameter, variable importance analysis revealed the relative significance of nutritional and metabolic indicators in predicting event risk in cancer patients receiving anthracycline treatment (Figure 2). Factors such as GAR and FBG emerged as the key predictors of MACE, along with RAR as a key predictor of all‐cause mortality.

### 3.3 Prognostic Values and Risk Stratification of GAR

**FIGURE 3:**
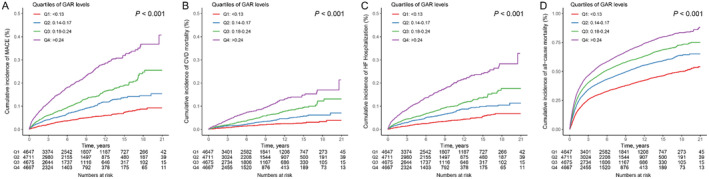
Kaplan–Meier survival analysis of glucose‐albumin ratio (GAR) for the outcome events in patients with cancer who were treated with anthracyclines. (A) major adverse cardiovascular events (MACE), (B) cardiovascular disease (CVD) mortality, (C) hospitalization due to heart failure (HHF), and (D) all‐cause mortality.

GAR was significantly associated with the risks of cardiovascular events. Figure 3 illustrates the results of the Kaplan–Meier survival analysis, showing that higher GAR was associated with a significantly higher risk of MACE, CVD death, HHF, and all‐cause death in cancer patients prescribed with anthracycline (all log‐rank *p* < 0.001).

When being examined in the fully adjusted model, compared to the lowest quartile (reference group), patients in the highest quartile of GAR had a significantly higher risk for MACE (HR, 2.23 [95% CI, 1.88–2.65]), as well as a higher risk of CVD death (HR, 2.56 [95% CI, 1.95–3.36]), HHF rehospitalization (HR, 2.11 [95% CI, 1.72–2.58]), and all‐cause mortality (HR, 1.72 [95% CI, 1.62–1.83]). Across increasing quartiles of GAR, the overall risk of events increased in a graded fashion (Table 2). Similar analysis and results were observed with quartiles of other metabolic and nutritional parameters (Table S2 **–** S5), while GAR demonstrated the highest HR for cardiovascular events consistent with the VIMP findings.

**TABLE 2:** HR (95% CIs) of end point events (major adverse cardiovascular event—cardiovascular mortality and hospitalization for heart failure) and all‐cause mortality according to quartiles of GAR in cancer patients treated with anthracyclines.

	Quartiles of GAR levels				*p*_trend_
	< 0.09	0.09–0.12	0.13–0.18	> 0.18	
MACE					
Crude	1.00 [Reference]	1.89 (1.58–2.25)	2.79 (2.35–3.30)	5.31 (4.53–6.22)	< 0.01
Model 1	1.00 [Reference]	1.56 (1.31–1.86)	2.02 (1.71–2.40)	3.25 (2.77–3.82)	< 0.01
Model 2	1.00 [Reference]	1.46 (1.23–1.74)	1.67 (1.41–1.99)	2.23 (1.88–2.65)	< 0.01
CVD‐related mortality					
Crude	1.00 [Reference]	1.84 (1.39–2.45)	3.09 (2.36–4.03)	5.39 (4.18–6.94)	< 0.01
Model 1	1.00 [Reference]	1.53 (1.16–2.04)	2.25 (1.72–2.94)	3.32 (2.57–4.29)	< 0.01
Model 2	1.00 [Reference]	1.44 (1.08–1.91)	1.94 (1.48–2.55)	2.56 (1.95–3.36)	< 0.01
Heart failure hospitalization					
Crude	1.00 [Reference]	1.88 (1.53–2.31)	2.70 (2.21–3.29)	5.45 (4.53–6.57)	< 0.01
Model 1	1.00 [Reference]	1.54 (1.25–1.89)	1.93 (1.58–2.36)	3.27 (2.71–3.95)	< 0.01
Model 2	1.00 [Reference]	1.44 (1.17–1.78)	1.57 (1.28–1.92)	2.11 (1.72–2.58)	< 0.01
All‐cause mortality					
Crude	1.00 [Reference]	1.45 (1.36–1.54)	1.83 (1.72–1.94)	2.34 (2.21–2.48)	< 0.01
Model 1	1.00 [Reference]	1.37 (1.29–1.45)	1.66 (1.56–1.76)	2.00 (1.88–2.12)	< 0.01
Model 2	1.00 [Reference]	1.28 (1.21–1.36)	1.45 (1.36–1.54)	1.72 (1.62–1.83)	< 0.01

*Note:* Data are presented as HR (95% CI) unless indicated otherwise; Model 1 was adjusted as age and sex; Model 2 was adjusted for age, sex, body mass index, estimated glomerular filtration rate, aspartate aminotransferase, hypertension, diabetes mellitus, atrial fibrillation, coronary artery disease, tumor type, renin‐angiensin system inhibitors use, statin use, cumulative dosing, and other classes of chemotherapy drugs.

Abbreviations: CI, confidence interval; CVD, cardiovascular disease; GAR, glucose‐to‐albumin ratio; HR, hazard ratio.

**FIGURE 4:**
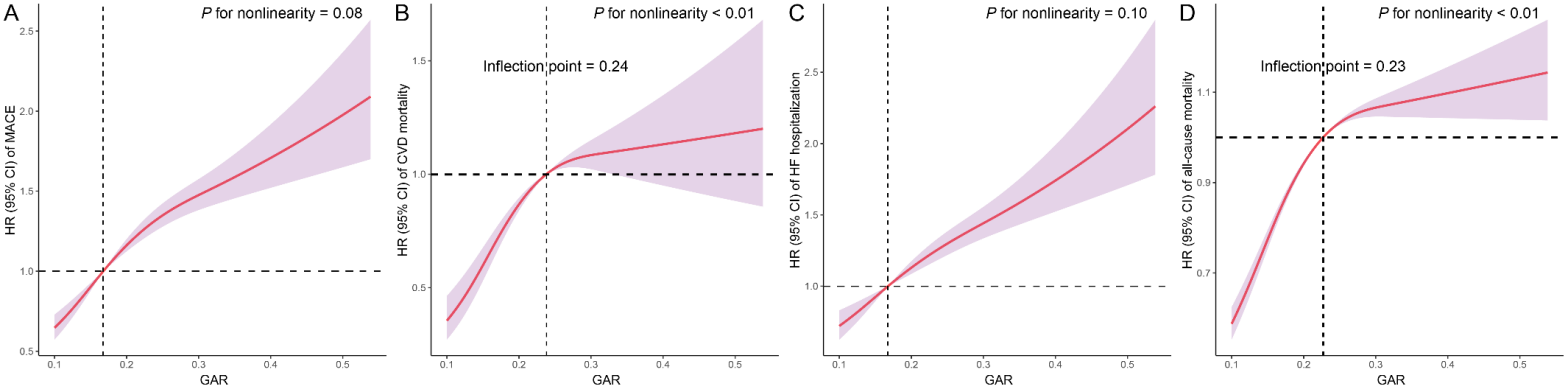
Restricted cubic spline curves of hazard ratio (with 95% confidence interval [CI]) of outcome events associated with the glucose‐albumin ratio (GAR). (A) major adverse cardiovascular events (MACE), (B) cardiovascular disease (CVD) mortality, (C) hospitalization due to heart failure (HHF), and (D) all‐cause mortality.

Restricted cubic spline (RCS) regression models highlighted that higher GAR levels are associated with increased risks of MACE (P for nonlinearity = 0.08, Figure 4A), CVD mortality (*p* for nonlinearity < 0.01, Figure 4B), HF hospitalization (P for nonlinearity = 0.10, Figure 4C), and all‐cause mortality (*p* for nonlinearity < 0.01, Figure 4D). Significant non‐linear relationships with critical inflection points at GAR values of approximately 0.23–0.24 were identified in the cases of CVD mortality and all‐cause mortality.

### 3.4 Subgroup Analysis

Subgroup analyses were conducted to explore potential effect modifiers that could influence the prognostic utility of GAR in cancer patients on anthracycline therapy. Factors such as age, sex, baseline comorbidities, treatment regimens, and anthracycline dosing were examined to assess their impact on the predictive performance of GAR. Results showed that higher GAR levels are consistently associated with increased risks of MACE in cancer patients treated with anthracyclines. This association was modulated by factors such as age, DM status, AF presence, cumulative dosing, and overweight and obesity (P for interaction < 0.01, Table 3), while sex and other comorbidities did not show significance.

**TABLE 3:** Stratified analyses of the associations between quartiles of GAR levels and the risks of primary endpoint events (heart failure and cardiovascular mortality) in cancer patients treated with anthracyclines.

Subgroups	N	Quartiles of GAR levels				*p*‐int
		< 0.13	0.14–0.17	0.18–0.24	> 0.24	
Age						
< 65 years old	10,560	1.00 [Reference]	1.45 (1.17–1.81)	1.74 (1.41–2.14)	2.2 (1.79–2.71)	< 0.01
≥ 65 years old	8140	1.00 [Reference]	1.6 (1.18–2.17)	1.79 (1.32–2.44)	2.97 (2.19–4.03)	
Sex						
Female	8686	1.00 [Reference]	1.59 (1.21–2.08)	1.8 (1.38–2.35)	2.53 (1.94–3.29)	0.40
Male	10,014	1.00 [Reference]	1.36 (1.08–1.72)	1.62 (1.29–2.03)	2.07 (1.65–2.59)	
Baseline HTN						
No	16,615	1.00 [Reference]	1.47 (1.22–1.78)	1.69 (1.40–2.04)	2.28 (1.89–2.75)	0.14
Yes	2085	1.00 [Reference]	1.24 (0.77–2.00)	1.41 (0.91–2.19)	1.74 (1.11–2.72)	
Baseline DM						
No	17,285	1.00 [Reference]	1.49 (1.24–1.78)	1.68 (1.41–2.01)	2.31 (1.93–2.75)	0.01
Yes	1415	1.00 [Reference]	0.6 (0.24–1.50)	0.64 (0.29–1.38)	0.74 (0.36–1.53)	
Baseline CAD						
No	18,390	1.00 [Reference]	1.45 (1.21–1.74)	1.66 (1.39–1.97)	2.24 (1.89–2.67)	0.34
Yes	310	1.00 [Reference]	2.06 (0.57–7.45)	2.29 (0.65–8.05)	2.48 (0.71–8.70)	
Baseline AF						
No	18,260	1.00 [Reference]	1.43 (1.19–1.71)	1.68 (1.41–2.01)	2.26 (1.90–2.71)	< 0.01
Yes	440	1.00 [Reference]	1.77 (0.86–3.63)	1.28 (0.66–2.50)	1.53 (0.78–3.00)	
Baseline CKD						
eGFR < 60	3208	1.00 [Reference]	1.47 (1.19–1.80)	1.64 (1.34–2.01)	2.41 (1.97–2.95)	0.10
eGFR ≥ 60	15,492	1.00 [Reference]	1.37 (0.97–1.95)	1.63 (1.17–2.26)	1.9 (1.37–2.64)	
Cumulative dose						
< 250 mg/m^2^	9078	1.00 [Reference]	1.53 (1.21–1.94)	1.87 (1.49–2.35)	2.56 (2.04–3.20)	0.03
≥ 250 mg/m^2^	3571	1.00 [Reference]	1.36 (1.04–1.78)	1.39 (1.06–1.81)	1.77 (1.35–2.31)	
Other chemotherapy						
No	10,367	1.00 [Reference]	1.45 (1.18–1.78)	1.74 (1.43–2.13)	2.23 (1.83–2.72)	0.14
Yes	8333	1.00 [Reference]	1.46 (1.04–2.05)	1.44 (1.02–2.03)	2.23 (1.59–3.14)	
Overweight and obesity						
No	11,125	1.00 [Reference]	1.65 (1.31–2.08)	1.6 (1.27–2.01)	2.12 (1.69–2.67)	< 0.01
Yes	7575	1.00 [Reference]	1.21 (0.91–1.60)	1.78 (1.37–2.30)	2.41 (1.86–3.12)	

*Note:* Data are presented as HR (95% CI) unless indicated otherwise; Analyses were adjusted for age, sex, body mass index, aspartate aminotransferase, HTN, DM, AF, CAD, eGFR, tumor type, renin‐angiotensin system inhibitors use, statin use, cumulative dosing, and other classes of chemotherapy drugs when they were not the strata variables.

Abbreviations: AF, atrial fibrillation; CAD, coronary artery disease; CKD, chronic kidney disease; DM, diabetes mellitus; eGFR, estimated glomerular filtration rate; GAR, glucose‐to‐albumin ratio; HTN, hypertension; *p‐int*, *p* for interaction.

Overall, the results are consistent with previous findings when analyzing for CVD mortality, HHF, and all‐cause mortality (Table S6 **–** S8). Notably, the association between quartiles of GAR levels and the risks of death (CVD and all‐cause mortality) was also influenced by CKD.

### 3.5 ROC Analysis of GAR in Different Time Points

**FIGURE 5:**
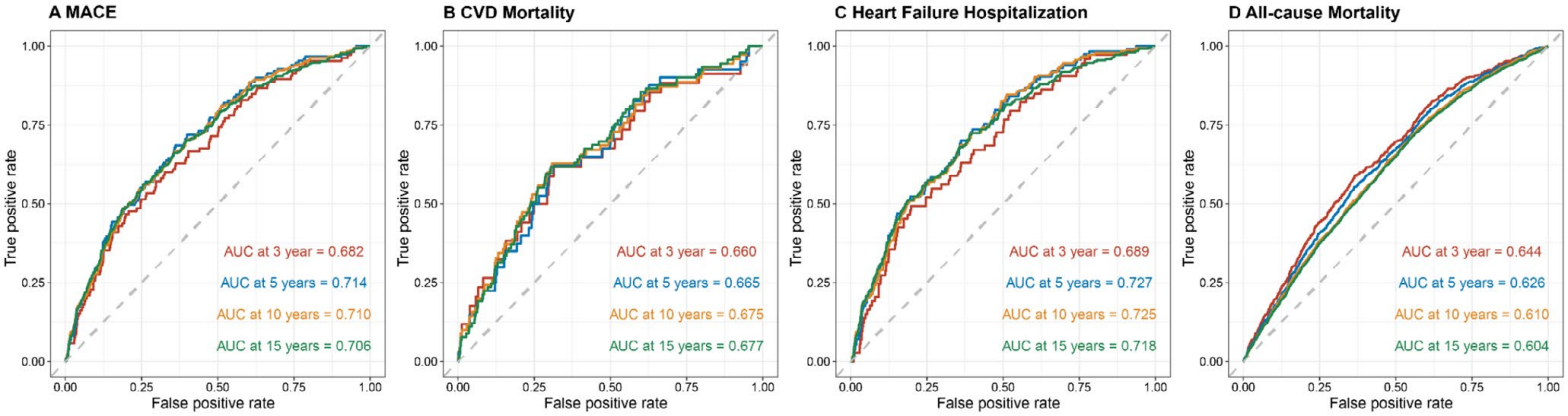
Receiver operator characteristic curve analysis of glucose‐albumin ratio (GAR) for the prediction of (A) major adverse cardiovascular events (MACE), (B) cardiovascular disease (CVD) mortality, (C) hospitalization due to heart failure (HHF), and (D) all‐cause mortality.

Receiver operating characteristic (ROC) analysis was performed at various time points, including 3‐, 5‐, 10‐, and 15‐year intervals, to evaluate the predictive performance of GAR in discerning cardiovascular risk and mortality risk in cancer patients prescribed with anthracycline. Results demonstrated reasonable predictive capability for MACE, with stable AUC values over longer time horizons. CVD mortality prediction demonstrates modest and slightly improving accuracy over time. The highest predictive performance is observed for heart failure hospitalization, with AUC values consistently above 0.70, particularly strong at the 5‐year interval (Figure 5).

### 3.6 Sensitivity Analysis

Table S9 showed the comparison of the AUC values for the prediction models before and after including GAR across different time points. Although not all were statistically significant, the inclusion of GAR in the fully adjusted models enhanced the risk prediction for MACE, CVD mortality, HHF, and all‐cause mortality, as indicated by the improvements in AUC values.

Table S10 presents the association between GAR quartiles and the risk of endpoint events after excluding participants who died within 1 year of follow‐up. Results showed that higher quartiles of GAR are significantly associated with increased risks of MACE, cardiovascular mortality, heart failure hospitalization, and all‐cause mortality (P for trend < 0.01). These findings remained consistent across different adjustment models, aligning with the results of the primary analyses and reinforcing the utility of GAR in risk stratification for these outcomes.

## 4 Discussion

Our findings reveal a significant association between higher GAR levels and worse overall cardiovascular outcomes among 18,700 cancer patients receiving anthracycline. GAR emerged as a significant predictor of MACE, CVD mortality, HHF, and all‐cause mortality. Higher GAR levels were consistently associated with increased risks across these outcomes, with hazard ratios peaking for MACE and CVD death. Our analyses indicated a stable and reliable predictive performance that underscores the utility of GAR in risk stratification for cardiovascular events and mortality among the high‐risk population.

Anthracyclines, while effective in treating various malignancies, are known to exert cardiotoxic effects, leading to an increased risk of cardiovascular complications such as cardiomyopathy, heart failure, and arrhythmias [15, 16]. Cardiovascular complications in cancer patients undergoing anthracycline therapy are a significant concern, necessitating thorough prognostic assessment tools. Identifying patients at heightened risk for these complications is crucial for implementing timely interventions and optimizing patient care [17].

The underlying mechanisms linking an elevated GAR to cardiovascular events may involve a combination of hyperglycemia‐induced vascular damage and hypoalbuminemia‐related impairment. Previous research has established the detrimental effects of hyperglycemia and hypoalbuminemia in patients with cardiovascular diseases and cancer [6, 7, 18]. Hyperglycemia can exacerbate oxidative stress and inflammation, leading to endothelial cell damage and atherosclerosis. The high glucose levels can lead to endothelial dysfunction, oxidative stress, and inflammation, all of which contribute to cardiovascular disease progression [19]. Concurrently, hypoalbuminemia may reflect a compromised nutritional state, reducing the ability to counteract these deleterious processes. Albumin serves as a carrier for various protective molecules, and its deficiency could diminish these protective effects, further escalating cardiovascular risk. The low serum albumin has been associated with increased morbidity and mortality due to its role in maintaining colloid oncotic pressure and binding and transport of various substances [20, 21].

Our study extends these findings by specifically focusing on the ratio of these two parameters—GAR in cancer patients treated with anthracyclines, highlighting its utility in identifying those at greater risk of cardiovascular complications. The clinical utility of GAR lies in its ability to serve as an easily accessible and cost‐effective biomarker for cardiovascular risk assessment. Monitoring GAR could enable clinicians to identify high‐risk patients early and implement preventive strategies, such as tighter glucose control or nutritional interventions, to mitigate their risk. This is particularly relevant in the context of anthracycline treatment, where timely cardiovascular monitoring and management are crucial due to the inherent cardiotoxicity of anthracycline.

In the context of cancer patients receiving anthracycline treatment, the interplay between metabolic disorder, malnutrition, and cardiotoxicity becomes paramount. While diabetes and prediabetes were common and were associated with an increased risk of heart failure and mortality in patients with cancer who received ACT [4, 7], exploring the role of GAR in conjunction with other biomarkers and clinical parameters could provide a more comprehensive risk stratification model. Investigating the interactions between anthracycline‐induced cardiotoxicity and metabolic and nutritional disorders will also pave the way for more targeted therapeutic strategies. Also, the lack of significant sex‐specific outcomes suggests that while biological sex is an important factor in many clinical contexts, GAR can be considered as a broadly applicable prognostic tool across different sexes.

Furthermore, the prognostic significance of GAR extends beyond traditional risk factors, offering additional insights into the complex interplay between cancer, anthracycline therapy, and cardiovascular health. Early identification of high‐risk patients can further optimize interventions, including aggressive cardiovascular monitoring, lifestyle modifications, and potentially dose adjustments or alternative treatment strategies [22, 23, 24]. Moreover, targeted interventions aimed at mitigating metabolic health and optimizing nutritional status may hold promise in attenuating cardiovascular complications in this vulnerable patient population [25]. By elucidating the mechanisms underlying cardiovascular risk in this patient population, our study contributes to a deeper understanding of the multifaceted nature of the cardiotoxicity associated with anthracycline treatment. Incorporating GAR assessment into risk stratification may enable more accurate identification of individuals at heightened cardiovascular risk.

## 5 Study Limitations

It is important to acknowledge several limitations of our study. First, the generalizability of our findings may be constrained to patients undergoing anthracycline‐based chemotherapy, limiting their applicability to other treatment regimens and patient populations. Additionally, the data was predominantly of Han ethnicity. This homogeneity prevented a detailed examination of racial differences, which is an important consideration given established racial disparities in cardiovascular health. Future investigations should aim to validate the prognostic value of GAR in more diverse cancer cohorts to evaluate its utility in clinical decision‐making. Second, the absence of echocardiographic data in the CDARS prevented the evaluation of the differential impact of systolic and diastolic function on cardiovascular outcomes. Third, CDARS, like many administrative databases, lacks systematic information on socioeconomic status, smoking status at the index date, and lifestyle factors, potentially introducing confounding variables. Also, the lack of comprehensive data on the physical activity levels of the patients in our dataset may impact the robustness of our predictions. Future research should aim to incorporate these detailed data to better understand its interaction with GAR and to refine cardiovascular risk stratification in this patient population. Moreover, while CDARS captures data for over 90% of the local population receiving care at public hospitals, it may not include information from patients treated at private hospitals or those who have immigrated. Lastly, despite employing multivariable adjustment techniques, the possibility of residual confounders influencing our results cannot be entirely ruled out. Addressing these limitations in future research will be crucial for validating the prognostic value of GAR and its role in guiding cardiovascular risk management in cancer patients undergoing anthracycline therapy.

## 6 Conclusion

In conclusion, the glucose to serum albumin ratio (GAR) emerges as a promising prognostic marker for cardiovascular outcomes in cancer patients undergoing anthracycline therapy. Elevated GAR serves as a robust indicator of increased cardiovascular risk, reflecting the interplay between metabolic and nutritional imbalances and cardiotoxicity. Further prospective studies are warranted to validate the utility of GAR as a prognostic tool, aiming to improve cardiovascular risk assessment and management in this vulnerable patient population.

## Author Contributions

**Iokfai Cheang:** conceptualization, data Curation, formal analysis, investigation, writing – original Draft. **Ying Li:** methodology, investigation, writing – review and editing. **Xu Zhu:** software, methodology, visualization. **Ziqi Chen:** data curation, visualization; **Qing‐Wen Ren:** data curation, investigation. **Mei‐Zhen Wu:** data curation, investigation. **Xin Xu:** data curation, investigation. **Hung‐Fat Tse:** project administration; **Kai‐Hang Yiu:** conceptualization, funding, validation, supervision, project administration. **Xinli Li:** visualization, resources, project administration.

## Ethics Statement

The University of Hong Kong institutional ethics review board and the West Cluster of HA approved this study (UW 21–270).

## Conflicts of Interest

The authors declare no conflicts of interest.

## Supporting information


**Table S1.** Definition of covariates.
**Table S2.** HR (95% CIs) of MACE according to quartiles of inflammatory/nutritional indicators in cancer patients treated with anthracyclines.
**Table S3.** HR (95% CIs) of CVD mortality according to quartiles of inflammatory/nutritional indicators in cancer patients treated with anthracyclines.
**Table S4.** HR (95% CIs) of HF hospitalization according to quartiles of inflammatory/nutritional indicators in cancer patients treated with anthracyclines.
**Table S5.** HR (95% CIs) of all‐cause mortality according to quartiles of inflammatory/nutritional indicators in cancer patients treated with anthracyclines.
**Table S6.** Stratified analyses of the associations between quartiles of GAR levels and the risks of cardiovascular mortality in cancer patients treated with anthracyclines.
**Table S7.** Stratified analyses of the associations between quartiles of GAR levels and the risks of heart failure hospitalization in cancer patients treated with anthracyclines.
**Table S8.** Stratified analyses of the associations between quartiles of GAR levels and the risks of all‐cause mortality in cancer patients treated with anthracyclines.
**Table S9.** Improvement of risk prediction by adding GAR to fully adjusted model.
**Table S10.** HR (95% CIs) of endpoint events (major adverse cardiovascular event—cardiovascular mortality and hospitalization for heart failure) and all‐cause mortality according to quartiles of GAR after excluding participants who died within 1 years of follow‐up in cancer patients treated with anthracyclines.

## Data Availability

The datasets used and/or analyzed during the current study are available from the corresponding author upon reasonable request.
