# Supplementary material for: Prognosis Prediction of Cardiovascular Event With Glucose‐Albumin Ratio on Patients With Cancer and Prescribed With Anthracycline
Source: Cancer Med. 2024 Dec 11;13(23):e70471. doi: 10.1002/cam4.70471 (PMC11632394; doi:10.1002/cam4.70471)
Supplement: Supplementary file 1 — Table S1. Definition of covariates. Table S2. HR (95% CIs) of MACE according to quartiles of inflammatory/nutritional indicators in cancer patients treated with anthracyclines. Table S3. HR (95% CIs) of CVD mortality according to quartiles of inflammatory/nutritional indicators in cancer patients treated with anthracyclines. Table S4. HR (95% CIs) of HF hospitalization according to quartiles of inflammatory/nutritional indicators in cancer patients treated with anthracyclines. Table S5. HR (95% CIs) of all‐cause mortality according to quartiles of inflammatory/nutritional indicators in cancer patients treated with anthracyclines. Table S6. Stratified analyses of the associations between quartiles of GAR levels and the risks of cardiovascular mortality in cancer patients treated with anthracyclines. Table S7. Stratified analyses of the associations between quartiles of GAR levels and the risks of heart failure hospitalization in cancer patients treated with anthracyclines. Table S8. Stratified analyses of the associations between quartiles of GAR levels and the risks of all‐cause mortality in cancer patients treated with anthracyclines. Table S9. Improvement of risk prediction by adding GAR to fully adjusted model. Table S10. HR (95% CIs) of endpoint events (major adverse cardiovascular event—cardiovascular mortality and hospitalization for heart failure) and all‐cause mortality according to quartiles of GAR after excluding participants who died within 1 years of follow‐up in cancer patients treated with anthracyclines. [file CAM4-13-e70471-s001.docx]

**Supplementary Materials**

**Table S1 - Definition of covariates.**

| **Entity** | **Definition of ICD9 codes** | **Definition of ICD10 codes** |
| --- | --- | --- |
| Atrial Fibrillation | 427.3 | I48 |
| Coronary Artery Disease | 410-414 | I20-I25 |
| Cerebrovascular disease | 430-438 | I60-I69 |
| Diabetes | 250 | E11 |
| Hypertension | 401-405 | I10 |
| Heart Failure | 402.01, 402.11, 402.91, 404.01, 404.03, 404.11, 404.13, 404.91, 404.93, 425.4-425.9, 428 | I11.0, I13.0, I13.2, I42.5, I42.7, I43, I50 |
| Haemorrhagic stroke | 430‐432 | I60-I62 |
| Hematologic diseases | 200-209 | C81-C96 |
| Ischaemic stroke | 433-434 | I63, I65, I66 |
| Malignancy | 140–239 | C00-C97 |
| Malignant neoplasm of breast | 174 | C50 |
| Malignant neoplasm of bladder | 188 | C67 |
| Metastasis | 196–199 | C78, C79 |

ICD: International Classification of Diseases

**Table S2.** HR (95% CIs) of MACE according to quartiles of inflammatory/nutritional indicators in cancer patients treated with anthracyclines.

|  | Quartiles of inflammatory/nutritional indicators | | | | *P*_trend_ |
| --- | --- | --- | --- | --- | --- |
|  | Quartile 1 | Quartile 2 | Quartile 3 | Quartile 4 |  |
| **NEU** | |  |  |  |  |
| Crude | 1.00 [Reference] | 1.24 (1.07-1.43) | 1.37 (1.18-1.58) | 1.63 (1.42-1.88) | <0.01 |
| Model 1 | 1.00 [Reference] | 1.04 (0.90-1.20) | 1.17 (1.01-1.36) | 1.39 (1.20-1.60) | <0.01 |
| Model 2 | 1.00 [Reference] | 1.04 (0.90-1.21) | 1.15 (0.99-1.34) | 1.24 (1.07-1.44) | <0.01 |
| **RDW** | |  |  |  |  |
| Crude | 1.00 [Reference] | 1.55 (1.35-1.78) | 1.96 (1.71-2.25) | 1.95 (1.68-2.25) | <0.01 |
| Model 1 | 1.00 [Reference] | 1.29 (1.12-1.49) | 1.65 (1.43-1.89) | 2.06 (1.78-2.38) | <0.01 |
| Model 2 | 1.00 [Reference] | 1.26 (1.10-1.45) | 1.44 (1.25-1.65) | 1.69 (1.45-1.96) | <0.01 |
| **FBG** |  |  |  |  |  |
| Crude | 1.00 [Reference] | 1.37 (1.15-1.63) | 2.34 (2.00-2.75) | 4.23 (3.64-4.92) | <0.01 |
| Model 1 | 1.00 [Reference] | 1.23 (1.03-1.47) | 1.77 (1.51-2.08) | 2.73 (2.35-3.18) | <0.01 |
| Model 2 | 1.00 [Reference] | 1.15 (0.97-1.37) | 1.47 (1.25-1.73) | 1.84 (1.56-2.17) | <0.01 |
| **HbA1c** |  |  |  |  |  |
| Crude | 1.00 [Reference] | 0.91 (0.78-1.07) | 1.48 (1.28-1.72) | 2.32 (2.03-2.64) | <0.01 |
| Model 1 | 1.00 [Reference] | 0.87 (0.74-1.02) | 1.22 (1.05-1.41) | 1.67 (1.46-1.91) | <0.01 |
| Model 2 | 1.00 [Reference] | 0.87 (0.74-1.02) | 1.04 (0.89-1.20) | 1.22 (1.05-1.41) | <0.01 |
| **ALB** |  |  |  |  |  |
| Crude | 1.00 [Reference] | 0.86 (0.75-0.97) | 0.68 (0.59-0.77) | 0.44 (0.38-0.51) | <0.01 |
| Model 1 | 1.00 [Reference] | 0.84 (0.74-0.95) | 0.75 (0.66-0.85) | 0.58 (0.50-0.68) | <0.01 |
| Model 2 | 1.00 [Reference] | 0.90 (0.79-1.03) | 0.83 (0.73-0.95) | 0.63 (0.54-0.73) | <0.01 |
| **NAR** |  |  |  |  |  |
| Crude | 1.00 [Reference] | 1.16 (1.00-1.34) | 1.45 (1.26-1.68) | 1.78 (1.54-2.05) | <0.01 |
| Model 1 | 1.00 [Reference] | 0.97 (0.84-1.13) | 1.19 (1.03-1.37) | 1.46 (1.26-1.68) | <0.01 |
| Model 2 | 1.00 [Reference] | 1.00 (0.86-1.16) | 1.14 (0.98-1.32) | 1.30 (1.12-1.50) | <0.01 |
| **RAR** |  |  |  |  |  |
| Crude | 1.00 [Reference] | 1.63 (1.40-1.90) | 2.53 (2.19-2.92) | 2.51 (2.16-2.91) | <0.01 |
| Model 1 | 1.00 [Reference] | 1.34 (1.15-1.56) | 1.90 (1.65-2.20) | 2.30 (1.98-2.68) | <0.01 |
| Model 2 | 1.00 [Reference] | 1.26 (1.08-1.46) | 1.71 (1.48-1.98) | 1.86 (1.59-2.17) | <0.01 |
| **HAR** |  |  |  |  |  |
| Crude | 1.00 [Reference] | 1.46 (1.24-1.71) | 1.97 (1.69-2.31) | 3.21 (2.77-3.73) | <0.01 |
| Model 1 | 1.00 [Reference] | 1.23 (1.04-1.45) | 1.41 (1.20-1.65) | 2.18 (1.87-2.53) | <0.01 |
| Model 2 | 1.00 [Reference] | 1.15 (0.98-1.36) | 1.19 (1.02-1.40) | 1.58 (1.35-1.85) | <0.01 |

Abbreviations: HR, hazard ratio; CI, confidence interval; NEU, neutrophil; RDW, red blood cell distribution width; FBG, fasting blood glucose; HbAlc, Hemoglobin A1c; ALB, albumin; NAR, neutrophil-to-albumin ratio; HAR, Hemoglobin A1c to albumin ratio.

Data are presented as HR (95% CI) unless indicated otherwise; Model 1 was adjusted as age, sex; Model 2 was adjusted as age, sex, body mass index, estimated glomerular filtration rate, aspartate aminotransferase, hypertension, diabetes mellitus, atrial fibrillation, coronary artery disease, tumor type, renin-angiotensin system inhibitors use, statin use, cumulative dosing, and other classes of chemotherapy drugs.

**Table S3.** HR (95% CIs) of CVD mortality according to quartiles of inflammatory/nutritional indicators in cancer patients treated with anthracyclines.

|  | Quartiles of inflammatory/nutritional indicators | | | | *P*_trend_ |
| --- | --- | --- | --- | --- | --- |
|  | Quartile 1 | Quartile 2 | Quartile 3 | Quartile 4 |  |
| **NEU** | |  |  |  |  |
| Crude | 1.00 [Reference] | 1.38 (1.09-1.75) | 1.45 (1.14-1.84) | 1.92 (1.53-2.42) | <0.01 |
| Model 1 | 1.00 [Reference] | 1.17 (0.92-1.48) | 1.25 (0.98-1.58) | 1.62 (1.29-2.04) | <0.01 |
| Model 2 | 1.00 [Reference] | 1.26 (0.99-1.61) | 1.29 (1.01-1.65) | 1.55 (1.22-1.96) | <0.01 |
| **RDW** | |  |  |  |  |
| Crude | 1.00 [Reference] | 1.59 (1.27-1.99) | 1.97 (1.58-2.46) | 2.18 (1.73-2.74) | <0.01 |
| Model 1 | 1.00 [Reference] | 1.32 (1.06-1.65) | 1.64 (1.32-2.06) | 2.30 (1.83-2.89) | <0.01 |
| Model 2 | 1.00 [Reference] | 1.28 (1.02-1.60) | 1.45 (1.15-1.81) | 1.84 (1.45-2.34) | <0.01 |
| **FBG** |  |  |  |  |  |
| Crude | 1.00 [Reference] | 1.08 (0.81-1.43) | 2.42 (1.89-3.09) | 3.94 (3.12-4.98) | <0.01 |
| Model 1 | 1.00 [Reference] | 0.97 (0.73-1.28) | 1.81 (1.42-2.32) | 2.54 (2.00-3.22) | <0.01 |
| Model 2 | 1.00 [Reference] | 0.91 (0.68-1.20) | 1.56 (1.21-2.00) | 1.95 (1.51-2.50) | <0.01 |
| **HbA1c** |  |  |  |  |  |
| Crude | 1.00 [Reference] | 0.90 (0.70-1.15) | 1.37 (1.09-1.72) | 1.92 (1.56-2.37) | <0.01 |
| Model 1 | 1.00 [Reference] | 0.86 (0.67-1.09) | 1.12 (0.89-1.40) | 1.38 (1.12-1.70) | <0.01 |
| Model 2 | 1.00 [Reference] | 0.86 (0.67-1.09) | 0.98 (0.78-1.23) | 1.05 (0.84-1.32) | 0.43 |
| **ALB** |  |  |  |  |  |
| Crude | 1.00 [Reference] | 0.93 (0.76-1.13) | 0.59 (0.47-0.73) | 0.43 (0.34-0.55) | <0.01 |
| Model 1 | 1.00 [Reference] | 0.91 (0.75-1.11) | 0.64 (0.52-0.80) | 0.56 (0.45-0.71) | <0.01 |
| Model 2 | 1.00 [Reference] | 0.99 (0.81-1.20) | 0.71 (0.57-0.88) | 0.61 (0.48-0.77) | <0.01 |
| **NAR** |  |  |  |  |  |
| Crude | 1.00 [Reference] | 1.31 (1.04-1.66) | 1.45 (1.14-1.84) | 2.06 (1.64-2.58) | <0.01 |
| Model 1 | 1.00 [Reference] | 1.11 (0.88-1.41) | 1.18 (0.93-1.50) | 1.68 (1.34-2.11) | <0.01 |
| Model 2 | 1.00 [Reference] | 1.22 (0.96-1.55) | 1.20 (0.94-1.54) | 1.59 (1.26-2.00) | <0.01 |
| **RAR** |  |  |  |  |  |
| Crude | 1.00 [Reference] | 1.49 (1.17-1.89) | 2.52 (2.02-3.15) | 2.51 (1.98-3.18) | <0.01 |
| Model 1 | 1.00 [Reference] | 1.23 (0.96-1.56) | 1.90 (1.52-2.39) | 2.31 (1.82-2.93) | <0.01 |
| Model 2 | 1.00 [Reference] | 1.17 (0.92-1.49) | 1.73 (1.38-2.17) | 1.84 (1.44-2.36) | <0.01 |
| **HAR** |  |  |  |  |  |
| Crude | 1.00 [Reference] | 1.42 (1.10-1.82) | 2.02 (1.58-2.57) | 2.85 (2.26-3.60) | <0.01 |
| Model 1 | 1.00 [Reference] | 1.21 (0.94-1.56) | 1.46 (1.14-1.86) | 1.95 (1.54-2.47) | <0.01 |
| Model 2 | 1.00 [Reference] | 1.14 (0.89-1.48) | 1.28 (1.00-1.63) | 1.49 (1.16-1.91) | <0.01 |

Abbreviations: HR, hazard ratio; CI, confidence interval; NEU, neutrophil; RDW, red blood cell distribution width; FBG, fasting blood glucose; HbAlc, Hemoglobin A1c; ALB, albumin; NAR, neutrophil-to-albumin ratio; HAR, Hemoglobin A1c to albumin ratio.

Data are presented as HR (95% CI) unless indicated otherwise; Model 1 was adjusted as age, sex; Model 2 was adjusted as age, sex, body mass index, estimated glomerular filtration rate, aspartate aminotransferase, hypertension, diabetes mellitus, atrial fibrillation, coronary artery disease, tumor type, renin-angiotensin system inhibitors use, statin use, cumulative dosing, and other classes of chemotherapy drugs.

**Table S4.** HR (95% CIs) of HF hospitalization according to quartiles of inflammatory/nutritional indicators in cancer patients treated with anthracyclines.

|  | Quartiles of inflammatory/nutritional indicators | | | | *P*_trend_ |
| --- | --- | --- | --- | --- | --- |
|  | Quartile 1 | Quartile 2 | Quartile 3 | Quartile 4 |  |
| **NEU** | |  |  |  |  |
| Crude | 1.00 [Reference] | 1.26 (1.06-1.50) | 1.43 (1.20-1.69) | 1.62 (1.37-1.91) | <0.01 |
| Model 1 | 1.00 [Reference] | 1.05 (0.89-1.25) | 1.22 (1.03-1.45) | 1.38 (1.16-1.63) | <0.01 |
| Model 2 | 1.00 [Reference] | 1.00 (0.84-1.19) | 1.15 (0.96-1.37) | 1.17 (0.98-1.39) | 0.02 |
| **RDW** | |  |  |  |  |
| Crude | 1.00 [Reference] | 1.50 (1.28-1.77) | 1.95 (1.66-2.28) | 1.79 (1.51-2.13) | <0.01 |
| Model 1 | 1.00 [Reference] | 1.25 (1.06-1.47) | 1.63 (1.39-1.92) | 1.90 (1.60-2.26) | <0.01 |
| Model 2 | 1.00 [Reference] | 1.23 (1.04-1.45) | 1.42 (1.20-1.67) | 1.60 (1.34-1.92) | <0.01 |
| **FBG** |  |  |  |  |  |
| Crude | 1.00 [Reference] | 1.52 (1.24-1.87) | 2.42 (2.00-2.94) | 4.51 (3.76-5.40) | <0.01 |
| Model 1 | 1.00 [Reference] | 1.37 (1.11-1.68) | 1.81 (1.49-2.20) | 2.87 (2.39-3.44) | <0.01 |
| Model 2 | 1.00 [Reference] | 1.28 (1.04-1.57) | 1.48 (1.22-1.80) | 1.79 (1.47-2.18) | <0.01 |
| **HbA1c** |  |  |  |  |  |
| Crude | 1.00 [Reference] | 0.95 (0.78-1.15) | 1.61 (1.35-1.92) | 2.64 (2.25-3.09) | <0.01 |
| Model 1 | 1.00 [Reference] | 0.90 (0.74-1.09) | 1.31 (1.10-1.56) | 1.88 (1.61-2.21) | <0.01 |
| Model 2 | 1.00 [Reference] | 0.91 (0.75-1.10) | 1.10 (0.92-1.32) | 1.33 (1.12-1.58) | <0.01 |
| **ALB** |  |  |  |  |  |
| Crude | 1.00 [Reference] | 0.75 (0.65-0.88) | 0.70 (0.61-0.82) | 0.42 (0.35-0.50) | <0.01 |
| Model 1 | 1.00 [Reference] | 0.74 (0.63-0.86) | 0.78 (0.67-0.91) | 0.56 (0.47-0.67) | <0.01 |
| Model 2 | 1.00 [Reference] | 0.79 (0.68-0.93) | 0.86 (0.74-1.00) | 0.60 (0.50-0.72) | <0.01 |
| **NAR** |  |  |  |  |  |
| Crude | 1.00 [Reference] | 1.14 (0.95-1.35) | 1.54 (1.30-1.82) | 1.77 (1.50-2.09) | <0.01 |
| Model 1 | 1.00 [Reference] | 0.95 (0.80-1.13) | 1.25 (1.06-1.48) | 1.45 (1.22-1.71) | <0.01 |
| Model 2 | 1.00 [Reference] | 0.93 (0.78-1.11) | 1.14 (0.96-1.36) | 1.23 (1.03-1.46) | <0.01 |
| **RAR** |  |  |  |  |  |
| Crude | 1.00 [Reference] | 1.65 (1.38-1.97) | 2.50 (2.11-2.96) | 2.47 (2.07-2.95) | <0.01 |
| Model 1 | 1.00 [Reference] | 1.34 (1.13-1.61) | 1.86 (1.57-2.20) | 2.27 (1.90-2.71) | <0.01 |
| Model 2 | 1.00 [Reference] | 1.25 (1.05-1.50) | 1.67 (1.41-1.99) | 1.87 (1.55-2.24) | <0.01 |
| **HAR** |  |  |  |  |  |
| Crude | 1.00 [Reference] | 1.45 (1.19-1.76) | 2.04 (1.69-2.46) | 3.47 (2.91-4.14) | <0.01 |
| Model 1 | 1.00 [Reference] | 1.21 (0.99-1.47) | 1.44 (1.19-1.74) | 2.31 (1.93-2.75) | <0.01 |
| Model 2 | 1.00 [Reference] | 1.13 (0.93-1.38) | 1.20 (0.99-1.45) | 1.62 (1.35-1.96) | <0.01 |

Abbreviations: HR, hazard ratio; CI, confidence interval; NEU, neutrophil; RDW, red blood cell distribution width; FBG, fasting blood glucose; HbAlc, Hemoglobin A1c; ALB, albumin; NAR, neutrophil-to-albumin ratio; HAR, Hemoglobin A1c to albumin ratio.

Data are presented as HR (95% CI) unless indicated otherwise; Model 1 was adjusted as age, sex; Model 2 was adjusted as age, sex, body mass index, estimated glomerular filtration rate, aspartate aminotransferase, hypertension, diabetes mellitus, atrial fibrillation, coronary artery disease, tumor type, renin-angiotensin system inhibitors use, statin use, cumulative dosing, and other classes of chemotherapy drugs.

**Table S5.** HR (95% CIs) of all-cause mortality according to quartiles of inflammatory/nutritional indicators in cancer patients treated with anthracyclines.

|  | Quartiles of inflammatory/nutritional indicators | | | | *P*_trend_ |
| --- | --- | --- | --- | --- | --- |
|  | Quartile 1 | Quartile 2 | Quartile 3 | Quartile 4 |  |
| **NEU** | |  |  |  |  |
| Crude | 1.00 [Reference] | 0.94 (0.89-0.99) | 1.05 (1.00-1.11) | 1.20 (1.13-1.26) | <0.01 |
| Model 1 | 1.00 [Reference] | 0.88 (0.83-0.93) | 1.00 (0.94-1.06) | 1.14 (1.08-1.20) | <0.01 |
| Model 2 | 1.00 [Reference] | 1.02 (0.96-1.08) | 1.11 (1.05-1.17) | 1.25 (1.19-1.33) | <0.01 |
| **RDW** | |  |  |  |  |
| Crude | 1.00 [Reference] | 1.30 (1.23-1.38) | 1.70 (1.60-1.80) | 2.44 (2.31-2.57) | <0.01 |
| Model 1 | 1.00 [Reference] | 1.21 (1.15-1.29) | 1.59 (1.51-1.69) | 2.48 (2.35-2.62) | <0.01 |
| Model 2 | 1.00 [Reference] | 1.20 (1.13-1.27) | 1.45 (1.37-1.53) | 1.95 (1.85-2.07) | <0.01 |
| **FBG** |  |  |  |  |  |
| Crude | 1.00 [Reference] | 1.19 (1.13-1.27) | 1.50 (1.42-1.59) | 1.81 (1.71-1.91) | <0.01 |
| Model 1 | 1.00 [Reference] | 1.15 (1.09-1.22) | 1.36 (1.28-1.44) | 1.55 (1.46-1.64) | <0.01 |
| Model 2 | 1.00 [Reference] | 1.11 (1.05-1.18) | 1.21 (1.15-1.29) | 1.39 (1.31-1.48) | <0.01 |
| **HbA1c** |  |  |  |  |  |
| Crude | 1.00 [Reference] | 0.86 (0.81-0.91) | 1.12 (1.06-1.18) | 1.17 (1.11-1.23) | <0.01 |
| Model 1 | 1.00 [Reference] | 0.84 (0.80-0.89) | 1.04 (0.98-1.10) | 1.03 (0.98-1.09) | <0.01 |
| Model 2 | 1.00 [Reference] | 0.86 (0.81-0.91) | 1.00 (0.95-1.05) | 0.95 (0.90-1.00) | 0.64 |
| **ALB** |  |  |  |  |  |
| Crude | 1.00 [Reference] | 0.75 (0.71-0.79) | 0.59 (0.56-0.62) | 0.48 (0.45-0.51) | <0.01 |
| Model 1 | 1.00 [Reference] | 0.75 (0.71-0.79) | 0.60 (0.57-0.64) | 0.52 (0.49-0.55) | <0.01 |
| Model 2 | 1.00 [Reference] | 0.82 (0.78-0.87) | 0.70 (0.67-0.74) | 0.61 (0.58-0.65) | <0.01 |
| **NAR** |  |  |  |  |  |
| Crude | 1.00 [Reference] | 0.93 (0.88-0.99) | 1.15 (1.09-1.22) | 1.36 (1.29-1.43) | <0.01 |
| Model 1 | 1.00 [Reference] | 0.88 (0.83-0.93) | 1.07 (1.02-1.14) | 1.28 (1.21-1.35) | <0.01 |
| Model 2 | 1.00 [Reference] | 1.01 (0.95-1.07) | 1.19 (1.12-1.25) | 1.35 (1.28-1.43) | <0.01 |
| **RAR** |  |  |  |  |  |
| Crude | 1.00 [Reference] | 1.41 (1.33-1.50) | 2.00 (1.89-2.12) | 2.92 (2.76-3.10) | <0.01 |
| Model 1 | 1.00 [Reference] | 1.32 (1.24-1.40) | 1.83 (1.72-1.94) | 2.84 (2.68-3.00) | <0.01 |
| Model 2 | 1.00 [Reference] | 1.27 (1.20-1.35) | 1.63 (1.53-1.73) | 2.17 (2.04-2.30) | <0.01 |
| **HAR** |  |  |  |  |  |
| Crude | 1.00 [Reference] | 1.22 (1.15-1.29) | 1.52 (1.44-1.61) | 1.86 (1.76-1.97) | <0.01 |
| Model 1 | 1.00 [Reference] | 1.15 (1.09-1.22) | 1.37 (1.29-1.45) | 1.64 (1.55-1.73) | <0.01 |
| Model 2 | 1.00 [Reference] | 1.09 (1.03-1.16) | 1.25 (1.18-1.33) | 1.38 (1.30-1.46) | <0.01 |

Abbreviations: HR, hazard ratio; CI, confidence interval; NEU, neutrophil; RDW, red blood cell distribution width; FBG, fasting blood glucose; HbAlc, Hemoglobin A1c; ALB, albumin; NAR, neutrophil-to-albumin ratio; HAR, Hemoglobin A1c to albumin ratio.

Data are presented as HR (95% CI) unless indicated otherwise; Model 1 was adjusted as age, sex; Model 2 was adjusted as age, sex, body mass index, estimated glomerular filtration rate, aspartate aminotransferase, hypertension, diabetes mellitus, atrial fibrillation, coronary artery disease, tumor type, renin-angiotensin system inhibitors use, statin use, cumulative dosing, and other classes of chemotherapy drugs.

**Table S6.** Stratified analyses of the associations between quartiles of GAR levels and the risks of cardiovascular mortality in cancer patients treated with anthracyclines.

| **Subgroups** | **N** | **Quartiles of GAR levels** | | | | ***p-int*** |
| --- | --- | --- | --- | --- | --- | --- |
|  |  | <0.13 | 0.14-0.17 | 0.18-0.24 | >0.24 |  |
| Age |  |  |  |  |  | <0.01 |
| <65 years old | 10560 | 1.00 [Reference] | 1.59 (1.12-2.25) | 2.11 (1.51-2.95) | 2.61 (1.87-3.65) |  |
| ≥65 years old | 8140 | 1.00 [Reference] | 1.26 (0.76-2.08) | 1.96 (1.22-3.16) | 3.21 (2.00-5.14) |  |
| Sex |  |  |  |  |  | 0.25 |
| Female | 8686 | 1.00 [Reference] | 1.24 (0.80-1.92) | 1.66 (1.09-2.53) | 2.37 (1.56-3.62) |  |
| Male | 10014 | 1.00 [Reference] | 1.53 (1.05-2.22) | 2.17 (1.52-3.10) | 2.70 (1.89-3.86) |  |
| Baseline HTN |  |  |  |  |  | 0.42 |
| No | 16615 | 1.00 [Reference] | 1.48 (1.09-2.00) | 1.90 (1.42-2.55) | 2.59 (1.93-3.47) |  |
| Yes | 2085 | 1.00 [Reference] | 1.14 (0.49-2.67) | 1.86 (0.87-3.99) | 2.04 (0.93-4.44) |  |
| Baseline DM |  |  |  |  |  | 0.02 |
| No | 17285 | 1.00 [Reference] | 1.49 (1.11-1.99) | 1.89 (1.43-2.50) | 2.61 (1.98-3.46) |  |
| Yes | 1415 | 1.00 [Reference] | 0.24 (0.04-1.53) | 0.92 (0.26-3.30) | 0.83 (0.25-2.78) |  |
| Baseline CAD |  |  |  |  |  | 0.07 |
| No | 18390 | 1.00 [Reference] | 1.42 (1.06-1.89) | 1.88 (1.43-2.47) | 2.60 (1.98-3.43) |  |
| Yes | 310 | 1.00 [Reference] | 3.01 (0.33-2.76) | 6.08 (0.69-5.32) | 2.68 (0.28-2.55) |  |
| Baseline AF |  |  |  |  |  | 0.03 |
| No | 18260 | 1.00 [Reference] | 1.43 (1.07-1.91) | 1.88 (1.43-2.49) | 2.55 (1.93-3.37) |  |
| Yes | 440 | 1.00 [Reference] | 1.48 (0.35-6.21) | 2.51 (0.72-8.76) | 2.17 (0.59-7.99) |  |
| Baseline CKD |  |  |  |  |  | <0.01 |
| eGFR <60 | 3208 | 1.00 [Reference] | 1.34 (0.96-1.86) | 1.70 (1.23-2.33) | 2.83 (2.07-3.86) |  |
| eGFR ≥60 | 15492 | 1.00 [Reference] | 1.68 (0.93-3.04) | 2.53 (1.45-4.44) | 2.14 (1.21-3.78) |  |
| Cumulative dosing |  |  |  |  |  | 0.18 |
| <250 mg/m^2^ | 9078 | 1.00 [Reference] | 1.57 (1.08-2.28) | 2.23 (1.56-3.19) | 2.78 (1.95-3.97) |  |
| ≥250 mg/m^2^ | 3571 | 1.00 [Reference] | 1.26 (0.81-1.96) | 1.54 (1.01-2.36) | 2.22 (1.45-3.40) |  |
| Other chemotherapy |  |  |  |  |  | 0.16 |
| No | 10367 | 1.00 [Reference] | 1.39 (1.00-1.94) | 2.06 (1.51-2.82) | 2.51 (1.83-3.44) |  |
| Yes | 8333 | 1.00 [Reference] | 1.48 (0.85-2.58) | 1.54 (0.88-2.68) | 2.60 (1.51-4.46) |  |
| Overweight and obesity | |  |  |  |  | 0.02 |
| No | 11125 | 1.00 [Reference] | 1.64 (1.15-2.35) | 1.85 (1.29-2.63) | 2.38 (1.66-3.40) |  |
| Yes | 7575 | 1.00 [Reference] | 1.13 (0.70-1.81) | 2.07 (1.36-3.16) | 2.85 (1.87-4.33) |  |

Abbreviations: *p-int*, *p* for interaction; GAR, glucose-to-albumin ratio; HTN: hypertension; HF: heart failure; CAD: coronary artery disease; CKD: chronic kidney disease; eGFR: estimated glomerular filtration rate.

Data are presented as HR (95% CI) unless indicated otherwise; Analyses were adjusted for age, sex, body mass index, estimated glomerular filtration rate, aspartate aminotransferase, hypertension, diabetes mellitus, atrial fibrillation, coronary artery disease, tumor type, renin-angiotensin system inhibitors use, statin use, cumulative dosing, and other classes of chemotherapy drugs when they were not the strata variables.

**Table S7.** Stratified analyses of the associations between quartiles of GAR levels and the risks of heart failure hospitalization in cancer patients treated with anthracyclines.

| **Subgroups** | **N** | **Quartiles of GAR levels** | | | | ***p-int*** |
| --- | --- | --- | --- | --- | --- | --- |
|  |  | <0.13 | 0.14-0.17 | 0.18-0.24 | >0.24 |  |
| Age |  |  |  |  |  | <0.01 |
| <65 years old | 10560 | 1.00 [Reference] | 1.37 (1.06-1.77) | 1.59 (1.24-2.02) | 2.05 (1.61-2.62) |  |
| ≥65 years old | 8140 | 1.00 [Reference] | 1.75 (1.22-2.51) | 1.72 (1.18-2.50) | 2.89 (1.99-4.20) |  |
| Sex |  |  |  |  |  | 0.23 |
| Female | 8686 | 1.00 [Reference] | 1.66 (1.21-2.28) | 1.91 (1.40-2.60) | 2.66 (1.95-3.63) |  |
| Male | 10014 | 1.00 [Reference] | 1.29 (0.98-1.70) | 1.36 (1.04-1.79) | 1.78 (1.36-2.33) |  |
| Baseline HTN |  |  |  |  |  | 0.11 |
| No | 16615 | 1.00 [Reference] | 1.43 (1.14-1.79) | 1.59 (1.27-1.99) | 2.16 (1.73-2.70) |  |
| Yes | 2085 | 1.00 [Reference] | 1.33 (0.78-2.28) | 1.32 (0.79-2.19) | 1.71 (1.03-2.86) |  |
| Baseline DM |  |  |  |  |  | <0.01 |
| No | 17285 | 1.00 [Reference] | 1.47 (1.19-1.82) | 1.61 (1.30-1.99) | 2.19 (1.78-2.71) |  |
| Yes | 1415 | 1.00 [Reference] | 0.66 (0.25-1.74) | 0.48 (0.21-1.13) | 0.66 (0.31-1.44) |  |
| Baseline CAD |  |  |  |  |  | 0.38 |
| No | 18390 | 1.00 [Reference] | 1.42 (1.15-1.76) | 1.57 (1.27-1.92) | 2.11 (1.72-2.59) |  |
| Yes | 310 | 1.00 [Reference] | 2.08 (0.45-9.59) | 1.60 (0.35-7.39) | 2.36 (0.52-10.67) |  |
| Baseline AF |  |  |  |  |  | <0.01 |
| No | 18260 | 1.00 [Reference] | 1.38 (1.11-1.71) | 1.58 (1.28-1.95) | 2.11 (1.71-2.61) |  |
| Yes | 440 | 1.00 [Reference] | 2.24 (0.99-5.05) | 1.26 (0.58-2.76) | 1.66 (0.76-3.61) |  |
| Baseline CKD |  |  |  |  |  | 0.23 |
| eGFR <60 | 3208 | 1.00 [Reference] | 1.51 (1.18-1.94) | 1.64 (1.28-2.09) | 2.27 (1.78-2.90) |  |
| eGFR ≥60 | 15492 | 1.00 [Reference] | 1.22 (0.82-1.80) | 1.31 (0.90-1.91) | 1.77 (1.23-2.55) |  |
| Cumulative dosing |  |  |  |  |  | 0.04 |
| <250 mg/m^2^ | 9078 | 1.00 [Reference] | 1.41 (1.07-1.86) | 1.61 (1.23-2.11) | 2.30 (1.77-2.99) |  |
| ≥250 mg/m^2^ | 3571 | 1.00 [Reference] | 1.46 (1.06-2.00) | 1.44 (1.05-1.98) | 1.79 (1.30-2.47) |  |
| Other chemotherapy |  |  |  |  |  | 0.28 |
| No | 10367 | 1.00 [Reference] | 1.46 (1.14-1.86) | 1.62 (1.28-2.05) | 2.14 (1.69-2.70) |  |
| Yes | 8333 | 1.00 [Reference] | 1.40 (0.93-2.09) | 1.39 (0.92-2.09) | 2.08 (1.38-3.13) |  |
| Overweight and obesity | |  |  |  |  | 0.01 |
| No | 11125 | 1.00 [Reference] | 1.60 (1.22-2.10) | 1.42 (1.08-1.87) | 1.95 (1.49-2.57) |  |
| Yes | 7575 | 1.00 [Reference] | 1.23 (0.88-1.70) | 1.77 (1.31-2.40) | 2.36 (1.74-3.19) |  |

Abbreviations: *p-int*, *p* for interaction; GAR, glucose-to-albumin ratio; HTN: hypertension; HF: heart failure; CAD: coronary artery disease; CKD: chronic kidney disease; eGFR: estimated glomerular filtration rate.

Data are presented as HR (95% CI) unless indicated otherwise; Analyses were adjusted for age, sex, body mass index, estimated glomerular filtration rate, aspartate aminotransferase, hypertension, diabetes mellitus, atrial fibrillation, coronary artery disease, tumor type, renin-angiotensin system inhibitors use, statin use, cumulative dosing, and other classes of chemotherapy drugs when they were not the strata variables.

**Table S8.** Stratified analyses of the associations between quartiles of GAR levels and the risks of all-cause mortality in cancer patients treated with anthracyclines.

| **Subgroups** | **N** | **Quartiles of GAR levels** | | | | ***p-int*** |
| --- | --- | --- | --- | --- | --- | --- |
|  |  | <0.13 | 0.14-0.17 | 0.18-0.24 | >0.24 |  |
| Age |  |  |  |  |  | <0.01 |
| <65 years old | 10560 | 1.00 [Reference] | 1.26 (1.15-1.38) | 1.45 (1.33-1.59) | 1.69 (1.54-1.85) |  |
| ≥65 years old | 8140 | 1.00 [Reference] | 1.37 (1.26-1.48) | 1.58 (1.46-1.71) | 2.03 (1.86-2.21) |  |
| Sex |  |  |  |  |  | 0.54 |
| Female | 8686 | 1.00 [Reference] | 1.28 (1.17-1.40) | 1.48 (1.36-1.62) | 1.75 (1.60-1.93) |  |
| Male | 10014 | 1.00 [Reference] | 1.26 (1.16-1.37) | 1.47 (1.35-1.59) | 1.77 (1.63-1.92) |  |
| Baseline HTN |  |  |  |  |  | 0.40 |
| No | 16615 | 1.00 [Reference] | 1.28 (1.20-1.36) | 1.44 (1.35-1.54) | 1.70 (1.59-1.81) |  |
| Yes | 2085 | 1.00 [Reference] | 1.29 (1.03-1.63) | 1.42 (1.15-1.76) | 1.74 (1.40-2.16) |  |
| Baseline DM |  |  |  |  |  | 0.12 |
| No | 17285 | 1.00 [Reference] | 1.28 (1.21-1.36) | 1.44 (1.35-1.53) | 1.7 (1.59-1.81) |  |
| Yes | 1415 | 1.00 [Reference] | 1.25 (0.68-2.31) | 1.62 (0.93-2.79) | 2.01 (1.19-3.40) |  |
| Baseline CAD |  |  |  |  |  | 0.29 |
| No | 18390 | 1.00 [Reference] | 1.28 (1.21-1.36) | 1.45 (1.37-1.54) | 1.72 (1.62-1.83) |  |
| Yes | 310 | 1.00 [Reference] | 1.13 (0.59-2.15) | 1.24 (0.66-2.31) | 1.62 (0.88-3.00) |  |
| Baseline AF |  |  |  |  |  | <0.01 |
| No | 18260 | 1.00 [Reference] | 1.29 (1.21-1.37) | 1.45 (1.36-1.54) | 1.72 (1.62-1.83) |  |
| Yes | 440 | 1.00 [Reference] | 1.07 (0.64-1.79) | 1.31 (0.83-2.07) | 1.55 (0.98-2.45) |  |
| Baseline CKD |  |  |  |  |  | <0.01 |
| eGFR <60 | 3208 | 1.00 [Reference] | 1.29 (1.21-1.38) | 1.44 (1.35-1.53) | 1.78 (1.67-1.91) |  |
| eGFR ≥60 | 15492 | 1.00 [Reference] | 1.10 (0.95-1.28) | 1.33 (1.16-1.54) | 1.34 (1.16-1.55) |  |
| Cumulative dosing |  |  |  |  |  | <0.01 |
| <250 mg/m^2^ | 9078 | 1.00 [Reference] | 1.33 (1.24-1.43) | 1.44 (1.34-1.55) | 1.73 (1.61-1.87) |  |
| ≥250 mg/m^2^ | 3571 | 1.00 [Reference] | 1.18 (1.06-1.32) | 1.44 (1.30-1.61) | 1.67 (1.49-1.87) |  |
| Other chemotherapy |  |  |  |  |  | 0.08 |
| No | 10367 | 1.00 [Reference] | 1.29 (1.19-1.40) | 1.47 (1.35-1.59) | 1.71 (1.57-1.86) |  |
| Yes | 8333 | 1.00 [Reference] | 1.24 (1.13-1.35) | 1.34 (1.22-1.47) | 1.63 (1.49-1.79) |  |
| Overweight and obesity | |  |  |  |  | 0.03 |
| No | 11125 | 1.00 [Reference] | 1.23 (1.14-1.33) | 1.45 (1.35-1.57) | 1.73 (1.60-1.87) |  |
| Yes | 7575 | 1.00 [Reference] | 1.37 (1.24-1.52) | 1.44 (1.30-1.59) | 1.70 (1.53-1.88) |  |

Abbreviations: *p-int*, *p* for interaction; GAR, glucose-to-albumin ratio; HTN: hypertension; HF: heart failure; CAD: coronary artery disease; CKD: chronic kidney disease; eGFR: estimated glomerular filtration rate.

Data are presented as HR (95% CI) unless indicated otherwise; Analyses were adjusted for age, sex, body mass index, estimated glomerular filtration rate, aspartate aminotransferase, hypertension, diabetes mellitus, atrial fibrillation, coronary artery disease, tumor type, renin-angiotensin system inhibitors use, statin use, cumulative dosing, and other classes of chemotherapy drugs when they were not the strata variables.

**Table S9.** Improvement of risk prediction by adding GAR to fully adjusted model.

|  | 3 years AUC (95% CI) | 5 years AUC (95% CI) | 10 years AUC (95% CI) | 15 years AUC (95% CI) |
| --- | --- | --- | --- | --- |
| **MACE** |  |  |  |  |
| Model* | 76.83 (71.96-81.70) | 79.29 (75.51-83.08) | 81.03 (78.26-83.80) | 79.25 (76.72-81.78) |
| Model + GAR | 77.39 (72.61-82.16) | 79.89 (76.18-83.60) | 81.45 (78.73-84.17) | 79.77 (77.28-82.25) |
| *P* value | 0.04 | <0.01 | 0.01 | <0.01 |
| **CVD Mortality** | |  |  |  |
| Model* | 73.15 (65.79-80.52) | 72.97 (65.84-80.10) | 74.60 (69.26-79.94) | 74.37 (69.58-79.17) |
| Model + GAR | 74.46 (67.25-81.67) | 74.15 (67.14-81.15) | 75.70 (70.52-80.89) | 75.40 (70.73-80.07) |
| *P* value | 0.10 | 0.08 | 0.02 | <0.01 |
| **Heart Failure Hospitalization** | |  |  |  |
| Model* | 81.08 (75.43-86.73) | 82.86 (78.77-86.95) | 84.49 (81.64-87.34) | 81.80 (79.00-84.59) |
| Model + GAR | 81.23 (75.63-86.83) | 83.19 (79.14-87.23) | 84.67 (81.83-87.50) | 82.15 (79.37-84.92) |
| *P* value | 0.46 | 0.04 | 0.14 | <0.01 |
| **All-cause Mortality** | |  |  |  |
| Model* | 72.58 (70.22-74.94) | 72.39 (70.55-74.23) | 72.48 (71.14-73.81) | 71.58 (70.43-72.74) |
| Model + GAR | 73.69 (71.39-75.98) | 73.30 (71.50-75.10) | 73.08 (71.76-74.40) | 72.16 (71.02-73.31) |
| *P* value | <0.01 | <0.01 | <0.01 | <0.01 |

CI, confidence interval; CV, cardiovascular; GAR, glucose-to-albumin ratio.

*Model was adjusted for age, sex, body mass index, estimated glomerular filtration rate, aspartate aminotransferase, hypertension, diabetes mellitus, atrial fibrillation, coronary artery disease, tumor type, renin-angiotensin system inhibitors use, statin use, cumulative dosing, and other classes of chemotherapy drugs.

**Table S10.** HR (95% CIs) of endpoint events (major adverse cardiovascular event—cardiovascular mortality and hospitalization for heart failure) and all-cause mortality according to quartiles of GAR after excluding participants who died within one years of follow-up in cancer patients treated with anthracyclines.

|  | Quartiles of GAR levels | | | | *P*_trend_ |
| --- | --- | --- | --- | --- | --- |
|  | <0.09 | 0.09-0.12 | 0.13-0.18 | >0.18 |  |
| **MACE** | |  |  |  |  |
| Crude | 1.00 [Reference] | 1.81 (1.50-2.19) | 2.78 (2.32-3.33) | 5.27 (4.44-6.24) | <0.01 |
| Model 1 | 1.00 [Reference] | 1.47 (1.22-1.78) | 1.97 (1.64-2.37) | 3.10 (2.61-3.68) | <0.01 |
| Model 2 | 1.00 [Reference] | 1.40 (1.16-1.69) | 1.68 (1.39-2.02) | 2.16 (1.80-2.60) | <0.01 |
| **Cardiovascular Mortality** | |  |  |  |  |
| Crude | 1.00 [Reference] | 1.68 (1.22-2.30) | 3.02 (2.25-4.06) | 5.48 (4.14-7.25) | <0.01 |
| Model 1 | 1.00 [Reference] | 1.35 (0.98-1.86) | 2.09 (1.55-2.82) | 3.13 (2.36-4.16) | <0.01 |
| Model 2 | 1.00 [Reference] | 1.29 (0.94-1.77) | 1.89 (1.39-2.55) | 2.46 (1.82-3.33) | <0.01 |
| **Heart Failure Hospitalization** | | |  |  |  |
| Crude | 1.00 [Reference] | 1.83 (1.47-2.28) | 2.75 (2.23-3.39) | 5.38 (4.42-6.54) | <0.01 |
| Model 1 | 1.00 [Reference] | 1.49 (1.20-1.86) | 1.95 (1.58-2.41) | 3.18 (2.61-3.88) | <0.01 |
| Model 2 | 1.00 [Reference] | 1.42 (1.14-1.76) | 1.62 (1.31-2.01) | 2.10 (1.69-2.60) | <0.01 |
| **All-Cause Mortality** | |  |  |  |  |
| Crude | 1.00 [Reference] | 1.43 (1.33-1.54) | 1.80 (1.67-1.93) | 2.34 (2.18-2.52) | <0.01 |
| Model 1 | 1.00 [Reference] | 1.30 (1.20-1.40) | 1.51 (1.40-1.63) | 1.77 (1.65-1.91) | <0.01 |
| Model 2 | 1.00 [Reference] | 1.23 (1.14-1.33) | 1.37 (1.27-1.47) | 1.58 (1.46-1.71) | <0.01 |

Abbreviations: HR, hazard ratio; CI, confidence interval; GAR, glucose-to-albumin ratio.

Data are presented as HR (95% CI) unless indicated otherwise; Model 1 was adjusted as age, sex; Model 2 was adjusted as age, sex, body mass index, estimated glomerular filtration rate, aspartate aminotransferase, hypertension, diabetes mellitus, atrial fibrillation, coronary artery disease, tumor type, renin-angiotensin system inhibitors use, statin use, cumulative dosing, and other classes of chemotherapy drugs.
